# MC1R: Front and Center in the Bright Side of Dark Eumelanin and DNA Repair

**DOI:** 10.3390/ijms19092667

**Published:** 2018-09-08

**Authors:** Viki B. Swope, Zalfa A. Abdel-Malek

**Affiliations:** Department of Dermatology, University of Cincinnati, 231 Albert Sabin Way, Cincinnati, OH 45267, USA; swopevk@ucmail.uc.edu

**Keywords:** human melanocytes, eumelanin, pheomelanin, photoprotection, melanocortin 1 receptor, paracrine factors, DNA repair

## Abstract

Melanin, the pigment produced by specialized cells, melanocytes, is responsible for skin and hair color. Skin pigmentation is an important protective mechanism against the DNA damaging and mutagenic effects of solar ultraviolet radiation (UV). It is acknowledged that exposure to UV is the main etiological environmental factor for all forms of skin cancer, including melanoma. DNA repair capacity is another major factor that determines the risk for skin cancer. Human melanocytes synthesize eumelanin, the dark brown form of melanin, as well as pheomelanin, which is reddish-yellow in color. The relative rates of eumelanin and pheomelanin synthesis by melanocytes determine skin color and the sensitivity of skin to the drastic effects of solar UV. Understanding the complex regulation of melanocyte function and how it responds to solar UV has a huge impact on developing novel photoprotective strategies to prevent skin cancer, particularly melanoma, the most fatal form, which originates from melanocytes. This review provides an overview of the known differences in the photoprotective effects of eumelanin versus pheomelanin, how these two forms of melanin are regulated genetically and biochemically, and their impact on the DNA damaging effects of UV exposure. Additionally, this review briefly discusses the role of paracrine factors, focusing on α-melanocortin (α-melanocyte stimulating hormone; α-MSH), in regulating melanogenesis and the response of melanocytes to UV, and describes a chemoprevention strategy based on targeting the melanocortin 1 receptor (MC1R) by analogs of its physiological agonist α-MSH.

## 1. The Evolution of Skin Pigmentation: An Important Adaptation to the Environment

Pigmentation is a complex trait that is regulated by a plethora of genes that are either extrinsic or intrinsic to melanocytes. These genes regulate melanocyte function and homeostasis by maintaining the proper expression and activity of receptors and their signaling pathways, transcription factors, melanogenic enzymes and structural proteins. In humans, the diversity of skin pigmentation evolved as an adaptation to the variations in geographic locations and the environment [1]. In particular, skin pigmentation is an adaptation to differences in the extent of solar ultraviolet radiation (UV) in different areas around the globe. Populations in equatorial regions that receive extensive UV have dark skin, which reduces the drastic effects of UV, such as skin cancer and folate degradation [1]. In contrast, populations of areas of higher latitudes with low UV evolved to have a light skin color that allows penetration of UV rays through the skin as an adaptation to synthesize optimal levels of vitamin D3. This adaptation is essential for the maintenance of bone health, the prevention of rickets, to insure reproductive capacity, and hence the preservation of the species [1].

## 2. Photoprotective Role of Melanin, Comparison of the Properties of Eumelanin Versus Pheomelanin

Melanocytes play a central role in conferring protection to the skin from the photocarcinogenic and photoaging effects of solar UV exposure [2,3,4]. It is unequivocal that solar UV is the main etiological factor for skin cancers, including melanoma, the most fatal [5,6,7]. By synthesizing the pigment melanin within specialized organelles, melanosomes, and transferring them to surrounding epidermal keratinocytes, melanocytes provide the skin with uniform pigmentation that reduces the genotoxic effects of UV [8]. Melanosomes localize in the perinuclear area of keratinocytes, forming “supranuclear caps” that reduce the extent of UV rays that penetrate through the epidermal layers and reach nuclear DNA, thereby shielding genomic DNA from excessive damage and genotoxicity [9,10]. It is established that the diversity of cutaneous pigmentation among humans determines the extent of photoprotection, and melanin content correlates inversely with the risk of sun-induced skin cancers and the extent of photoaging [11,12].

Human melanocytes, regardless of ethnicity or phototype, synthesize two main forms of melanin: eumelanin, which is dark brown/black in color, and pheomelanin, which is reddish-yellowish [13]. Chemical analysis of eumelanin and pheomelanin in human skin ex vivo and in primary melanocyte cultures derived from donors with different skin pigmentation revealed that eumelanin is the main determinant of the extent of pigmentation, and correlates directly with total melanin content [14,15]. Eumelanin is more stable and less prone to photodegradation than pheomelanin [16,17,18]. In dark skin, melanosomes enriched with eumelanin persist in keratinocytes throughout the epidermal layers [19,20]. In contrast, in lightly pigmented skin, melanosomes with low eumelanin content are degraded, and only “melanin dust”, presumed to be the degradation product of pheomelanin, is evident in the epidermis. Pheomelanin contains the amino acid cysteine, which gives it its distinctive red-yellow color [21]. The sulfhydryl bonds in cysteine are prone to oxidation, which reduces the stability of pheomelanin. Additionally, eumelanin and pheomelanin differ in their ability to quench reactive oxygen species (ROS) [22]. Eumelanin, but not pheomelanin, is highly efficient as a scavenger of ROS. Due to the significant clinical implications of the differences between eumelanin and pheomelanin on the sensitivity to UV exposure, risk for skin cancer and photoaging, there is great interest in investigating in depth their impact on the response of melanocytes to solar UV.

The photoprotective role of pigmentation was demonstrated in an in vivo study comparing the extent of UV-induced DNA damage in the skin of subjects with different skin pigmentation and ethnic origin [23]. The results showed an inverse correlation between melanin content and the levels of cyclobutane pyrimidine dimers (CPD), the major form of DNA photoproducts. In vitro experiments comparing the responses of human melanocyte cultures derived from donors with different skin phototypes and different total melanin and eumelanin contents to the same doses of UV confirmed the inverse relationship between eumelanin content and the generation of CPD [24].

## 3. Oxidative Stress, Impact of Eumelanin Versus Pheomelanin

There is increasing evidence that oxidative stress contributes to the genetic instability of melanocytes, and that melanoma is an oxidative-stress driven cancer [25,26]. Studies on the physico-chemical properties of eumelanin and pheomelanin concluded that eumelanin is superior to pheomelanin in reducing ROS. Additionally, stimulation of pheomelanin synthesis was reported to be accompanied by reduction in the levels of glutathione, the first-line-of-defense antioxidant [27]. This might be due to consumption of cysteine during pheomelanin synthesis, making it less available for the synthesis of glutathione. Additionally, glutathione itself might be utilized for the synthesis of 5-*S*-gluthatione-l-DOPA, a reaction catalyzed by the enzyme tyrosinase, and then converted to 5-*S*-l-cysteinyl-glycine-l-DOPA by γ-glutamyl transpeptidase, and finally to 5-*S*-l-cysteinyl-l-DOPA and glycine [28]. It was found that dysplastic nevi, a known risk factor for melanoma, have higher levels of pheomelanin, produce more ROS, and have greater oxidative DNA damage than melanocytes from normal skin of the same donor [27,29]. The pro-oxidant effect of pheomelanin was confirmed by the demonstration that it depletes glutathione, as well as NADPH, and induces the auto-oxidation of melanin precursors [30]. Further support for the role of pheomelanin in oxidative stress came from the report that in human melanocytes, the levels and activity of the antioxidant enzyme catalase correlate inversely with pheomelanin content, and melanocytes with a low melanin (i.e., low eumelanin) content are more sensitive to treatment with a pro-oxidant relative to their counterparts with high melanin content [31].

Additionally, an earlier study found that in mice expressing the activating *BRAF^v600E^* mutation, the most common driver mutation in melanoma [32], those with yellow coat color (that only synthesize pheomelanin due to loss-of-function mutation in *mc1r*) had the highest levels of oxidative DNA and lipid damage than mice with black coat color, or even albino mice that are totally devoid of pigment [33]. Surprisingly, in the absence of any exogenous chemical carcinogen or exposure to UV, the *BRAF^v600E^* mutant yellow mice spontaneously developed melanoma tumors [33]. The authors hypothesized that the pro-oxidant and oncogenic effects of pheomelanin might be attributed to (i) generation of ROS by pheomelanin itself, or (ii) depletion of antioxidant defenses by pheomelanin synthesis, thereby increasing the vulnerability of melanocytes to cellular ROS [34]. The presence of sulfur in pheomelanin makes it more likely to be involved in the generation of ROS than eumelanin. Although hydroxyl radicals produced by pheomelanin do not travel beyond 10 Å, they can nevertheless overwhelm the antioxidant capacity of melanocytes. Such an astounding oncogenic effect of pheomelanin is not clear in humans, and unlike yellow mice that exclusively synthesize pheomelanin, humans with red hair due to loss-of-function *MC1R* variants still synthesize low levels of eumelanin, in addition to pheomelanin [15,35]. Despite these species differences, the results reported by Mitra et al. [33] are significant in that they demonstrate the detrimental effect of pheomelanin, in the absence of the protective effect of eumelanin, on the susceptibility of melanocytes expressing *BRAF^v600E^*, to malignant transformation. It is noteworthy that this activating *BRAF* mutation is commonly expressed in nevi, some of which can be precursors to melanoma [36].

An intriguing observation was the continuous generation of CPD in mouse skin irradiated with ultraviolet A (UVA) for hours after the cessation of irradiation [37]. These latent CPD were coined “dark CPD”. Interestingly, the levels of immediate, as well as dark CPD were twice as high in the skin of *recessive yellow* (*mc1r^e/e^*) mice that synthesize only pheomelanin than in wild type black mice, indicating the poor ability of pheomelanin to shield UVA rays. The role of pheomelanin synthesis in the formation of these DNA photoproducts was further confirmed by the finding that incubation of plasmid DNA with 5-*S*-cysteinyl-DOPA, an intermediate of the pheomelanin synthetic pathway, resulted in the generation of CPD without any UV exposure. It is known that UVA induces indirect damage to DNA by increasing the generation of ROS [38,39]. Dark CPD were caused by the generation of superoxide and nitric oxide that excite an electron in degradation products of melanin, creating a quantum triplet state and energy transfer to DNA [37]. Since increased DNA damage can reduce the efficiency of DNA repair and increase the chance for mutagenesis, dark CPD can potentially overwhelm the DNA repair capacity of melanocytes, and contribute to somatic mutations that drive malignant transformation. It will be important to confirm the induction of dark CPD in human melanocytes, and to investigate if their levels depend on the relative eumelanin and pheomelanin contents. Such studies will define the role of ROS and nitrogen species, as well as the pro-oxidant effects of pheomelanin, in the genotoxic effects of UV that drive malignant transformation of human melanocytes to melanoma.

## 4. Genetic and Biochemical Regulation of Eumelanin vesus Pheomelanin Synthesis

**Figure 1:**
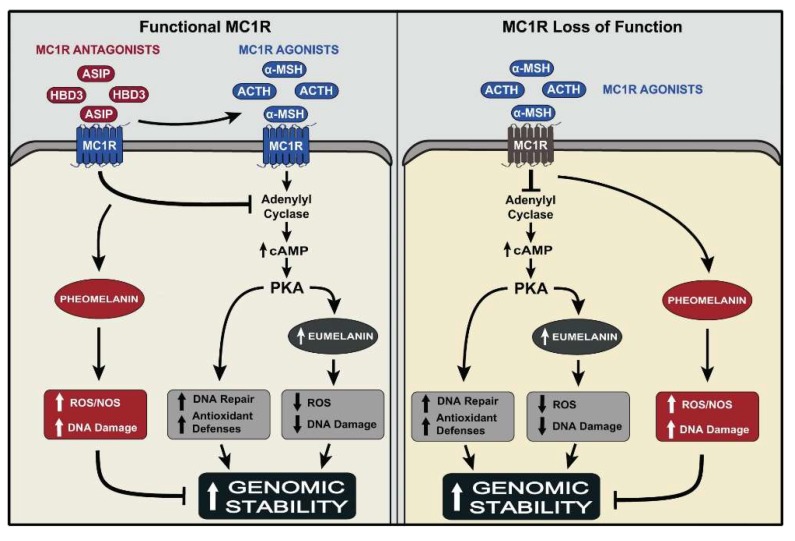
Summary of the effects of the MC1R agonists α-MSH and ACTH and the antagonists ASIP and HBD3 on functional MC1R, and the impact of loss of function of MC1R on these effects. Activation of the MC1R, expressed on the cell membrane of melanocytes by either agonist, α-MSH or ACTH activates the cAMP pathway, leading to increased eumelanin synthesis, which quenches reactive oxygen species (ROS) and reduces the generation of DNA damage upon UV exposure. Activation of the cAMP pathway also enhances the DNA repair and antioxidant capacities of melanocytes. The cumulative outcome of these effects is maintenance of genomic stability of melanocytes. Treatment of melanocytes with either ASIP or HBD3 antagonizes the effects of α-MSH, and are therefore expected to reduce genomic stability. Expression of loss-of-function-MC1R disrupts the signaling of the agonist-bound receptor, thereby inhibiting the synthesis of eumelanin, allowing only the synthesis of pheomelanin, which increases the generation of ROS and NOS, and allows for increased UV-induced DNA damage. Additionally, lack of signaling of the α-MSH-bound MC1R via the cAMP pathway inhibits the activation of antioxidant and DNA repair pathways, leading to reduced genomic stability of UV-irradiated melanocytes, and increased risk for malignant transformation to melanoma. Upward arrows: increase in the effect; T bar: blocking the effect.

The significance of eumelanin versus pheomelanin in determining skin pigmentation and the response to UV sparked the interest in elucidating the genetic and biochemical regulation of synthesis of these two forms of melanin by melanocytes. It has been known for decades that the *extension* locus in mice, which codes for the melanocortin 1 receptor (mc1r), is a central regulator of eumelanin synthesis [40,41]. The mc1r is a membrane-bound G_s_ protein-coupled receptor expressed on melanocytes [42]. Activation of the mc1r by binding of its physiological agonist α-melanocyte stimulating hormone (α-melanocortin; α-MSH) increases the synthesis of eumelanin in a cAMP-dependent manner (Figure 1) [40,43,44]. The *recessive yellow* mutation in *extension*, a frame-shift mutation that causes loss of function of the mc1r, results in a yellow coat color in mice, due to exclusive synthesis of pheomelanin and failure of melanocytes to synthesize eumelanin [41,43]. Accordingly, pheomelanin synthesis was proposed to be a default pathway: when melanocytes fail to synthesize eumelanin, they can still produce pheomelanin. This notion was supported by the biochemical findings that eumelanin synthesis requires high levels and activity of tyrosinase, the rate-limiting enzyme of melanin synthesis, as well as high levels of tyrosinase related (Tyrp)-1 and -2 (dopachrome tautomerase) [45,46]. In contrast, pheomelanin synthesis proceeds in the presence of low levels and activity of tyrosinase, and in the absence of Tyrp-1 and Tyrp-2. In human melanocytes, eumelanin synthesis is regulated similarly to mouse melanocytes by activation of MC1R by α-MSH. Treatment of human melanocytes with the potent melanocortin analog [Nle^4^, D-Phe^7^]-α-MSH (NDP-MSH), or treatment of mice with agents that increase the levels of cAMP, e.g., forskolin, results in increased eumelanin synthesis [47,48]. In humans, loss-of-function allelic variants of the *MC1R* are strongly associated with red hair color and fair skin, due to inhibition of eumelanin synthesis [35,49,50]. Additionally, mutations in the *pro-opiomelanocortin* (*POMC*) gene, which codes for the precursor of α-MSH, is associated with red hair phenotype, in addition to metabolic abnormalities [51]. The human MC1R recognizes both α-MSH and the structurally-related adrenocorticotropic hormone (ACTH) as agonists, and binds both ligands with the same affinity [52,53]. This explains the hyperpigmentation associated with over-production of ACTH, as in Addison’s disease [54]. Collectively, these results lend strong support to the role of MC1R, its agonists, and its signaling pathway in regulating the synthesis of eumelanin.

The main physiological antagonist for the mc1r is agouti signaling protein (ASIP), which is expressed in the mouse hair follicles in a temporal fashion, resulting in agouti phenotype, characterized by hairs with dark (eumelanin-containing) bands, interrupted by a yellow (pheomelanin-containing) band [55,56,57]. Mutations that cause overproduction of ASIP result in a yellow coat color of mice, a pigmentary phenotype similar to that caused by the *recessive yellow* mutation in *mc1r*. The human *Agouti* gene was cloned in human skin, and its product was shown to function as an inverse agonist of MC1R in human melanocytes [58,59]. Treatment of cultured human melanocytes with human ASIP resulted in displacement of α-MSH from MC1R, and abrogation of α-MSH-induced increase in cAMP levels and tyrosinase activity (Figure 1) [59]. Therefore, the MC1R/α-MSH/ASIP axis functions in human epidermal melanocytes, as in mouse follicular melanocytes, to regulate the eumelanin/pheomelanin switch.

Another physiological modulator of mc1r activity is human beta defensin 3 (HBD3), best known for its antimicrobial effects, and is synthesized by keratinocytes [60,61]. A deletion mutation in *CBD103*, the ortholog of *HBD3,* was first reported to result in a black coat color in dogs [62]. It was proposed that this mutation results in black pigmentation in dogs and transgenic mice via competing with, and inhibiting the binding of ASIP to the mc1r. We found that HBD3 acts as an antagonist of the human MC1R, blocking the effects of α-MSH on cAMP and tyrosinase activity in human melanocytes (Figure 1) [63]. Collectively, these studies underscore the significance of the MC1R, and defines its physiological agonists and antagonists and their function in regulating eumelanin and pheomelanin synthesis by human melanocytes.

In addition to *MC1R*, *agouti*, and *HBD3,* there are other genes that contribute to the regulation of eumelanin and pheomelanin synthesis. For example, *mahogany* and *mahoganoid* function as negative modifiers of *agouti* in mice, inhibiting its effects, thereby increasing eumelanin synthesis. Both *mahogany* and *mahoganoid* are thought to be downstream of *agouti* and upstream of *mc1r* [64]. The *pink eyed-dilution* (*p*) gene, which codes for a protein with 12 membrane spanning domains, is thought to be a melanosomal transmembrane protein that is involved in melanosome biogenesis, and functions as an anion transporter that regulates melanosomal pH, and the stability of tyrosinase-Tyrp-1 and Tyrp-2 complex, which is required for eumelanin synthesis [65]. Melanosomal pH is important in regulating eumelain/pheomelanin synthesis and neutral pH favors the synthesis of eumelanin [66]. Mutations in *p* gene reduce total melanin, mainly eumelanin content, and are the underlying cause of oculocutaneous albinism type-2. *Slc7a11* gene encodes the plasma membrane cysteine/glutamate exchanger Xct [67]. The recessive *sut* mutation in *Slc7a11* markedly reduces the synthesis of pheomelanin due to reduction in transport of extracellular cystine, which also leads to diminished levels of glutathione and ability to overcome oxidative stress.

## 5. *MC1R*, A Major Regulator of Human Pigmentation and a Melanoma Susceptibility Gene

The gene that is central to the regulation of human pigmentation is the *MC1R*. Epidemiological studies in different human populations concluded that *MC1R* is very highly polymorphic, and its many allelic variants account to a large extent for the diversity of human pigmentation [68,69,70]. Given the significance of the MC1R in regulating eumelanin synthesis, the *MC1R* genotype is expected to determine the sensitivity of melanocytes, and thereby the skin, to solar UV exposure. The consensus wild type *MC1R* is predominant in African countries, where dark skin pigmentation is mostly needed to mitigate the photodamaging effects of the equatorial sun [68,69,70]. Variants of *MC1R* are mostly prevalent in Northern latitudes, and a few, mainly R151C, R160W, and D294H, are strongly associated with red hair and fair skin phenotype [49,71]. These variants result in loss of function of the MC1R, inhibiting signaling of the α-MSH-bound receptor [35,50]. The resulting pigmentary phenotype is associated with poor tanning ability and increased risk for melanoma [5].

The *MC1R* is a *bona fide* melanoma predisposition gene [72,73]. Twenty-four percent of melanoma patients carry *MC1R* loss-of-function variants, and although redheads who express two *MC1R* loss-of-function alleles comprise only 1–2% of the worldwide population, they represent 16% of all melanoma patients [74,75]. A seminal finding was that MC1R regulates DNA repair and antioxidant pathways in melanocytes [35,76,77,78,79,80]. We and others reported that human melanocytes expressing functional MC1R respond to α-MSH treatment with increased efficiency of repair of UV-induced DNA photoproducts, reduced hydrogen peroxide generation and increased expression of antioxidant enzymes, such as catalase and hemeoxygenase-1, γ-glutamylcysteine synthase (γ-GCS), glutathione-*S*-transferase Pi (GSTPi), Peroxiredoxin 1 (PRX1), 8-oxoguanine DNA glycosylase (OGG1) and apurinic apyrimidinic endonuclease 1 (APE-1/Ref-1) (Figure 1). These effects of α-MSH can be mimicked by agents that activate the cAMP pathway, e.g., Forskolin, suggesting the significance of this pathway in maintaining the genomic stability of melanocytes [35,48,81]. Loss-of-function variants of *MC1R* not only inhibit the synthesis of eumelanin, but also compromise the DNA repair and antioxidant capacities of melanocytes (Figure 1) [35]. These findings were corroborated by the observation that *recessive yellow* mice, which expressed loss-of-function *mc1r* together with the somatic *BRAF^v600E^* mutation, had a markedly higher levels of oxidative DNA damage and lipid peroxidation than their counterparts that expressed wild type *mc1r* [33]. Further support for the role of *MC1R* in determining the extent of UV-induced genotoxicity and mutagenesis came from a recent report that loss-of-function *MC1R* variants increase the risk of melanocytes to acquire UV signature mutations that promote malignant transformation to melanoma [82].

Based on these findings, it can be concluded that expression of functional MC1R protects melanocytes against the genotoxic effects of UV by three mechanisms: (i) activation of DNA repair pathways (nucleotide excision and base excision repair pathways); (ii) inhibition of generation of reactive oxygen species, activation of antioxidant enzymes, and up regulation of expression of antioxidant genes; and (iii) increase in eumelanin synthesis (Figure 1) [35,76,78,79,80]. The first two mechanisms represent an early and immediate response to UV to prevent genomic instability of melanocytes, and the third is a latent response, which protects against the genotoxic effects of subsequent UV exposure.

## 6. Paracrine Factors as Regulators of Melanogenesis and the Response of Melanocytes to UV

In human skin, melanocytes differ from epidermal keratinocytes and dermal fibroblasts in their longevity in the skin and very limited proliferative capacity. Melanocytes survive in the epidermis for decades, and only proliferate upon injury in response to insult from the environment or its microenvironment [83]. Melanocytes are also resistant to apoptosis [84]. These properties make these cells prone to accumulate UV-induced somatic mutations over time due to repetitive sun exposure, which might culminate in malignant transformation to melanoma. It is established that melanocyte homeostasis is maintained by a network of paracrine factors, many of which are up regulated in expression by exposure to UV. The symbiotic relationship between melanocytes and keratinocytes has different levels. It involves the transfer of melanin-containing melanosomes from melanocytes to keratinocytes, to confer photoprotection to the entire epidermis [10]. It also includes the synthesis and secretion by keratinocytes of a wide array of factors that maintain the homeostasis of melanocytes and modulate their response to UV [85,86,87,88,89,90,91,92]. Keratinocytes and melanocytes synthesize POMC and process it to the bioactive peptides α-MSH, ACTH and β-endorphin [85,92,93]. The synthesis of POMC is increased in the epidermis in response to UV exposure [94,95]. Endothelin-1 is synthesized by keratinocytes, and is a potent mitogen for melanocytes in vitro [89,96,97]. We reported that endothelin-1 and α-MSH, together with the keratinocyte-derived mitogen basic fibroblast growth factor (bFGF), interact synergistically to support the proliferation of human melanocytes in vitro [98]. Importantly, endothelin-1, similar to α-MSH, enhances repair of DNA photoproducts and reduces apoptosis of UV-irradiated human melanocytes [76,99]. These results suggest that the paracrine factors α-MSH and endothelin-1 function as “survival factors” that enable melanocytes to overcome the stress imposed by UV exposure, allowing them to survive with genomic stability. Importantly, endothelin-1 compensates for the inability of melanocytes expressing loss-of-function MC1R to respond to α-MSH with modulating the DNA damage response to UV [99]. This provides an additional line of defense against UV-induced genotoxicity, and reduces the risk of these vulnerable melanocytes to transform to melanoma. Keratinocytes synthesize vitamin D3, and its active form, 1,25(OH)_2_ vitamin D3, increases the DNA repair capacity of UV-irradiated keratinocytes and melanocytes [100,101]. Consistent with these results, targeted deletion of vitamin D receptor gene in mouse melanocytes compromised melanin content, and increased the levels of CPD induced by UV exposure [102]. Importantly, all three paracrine factors, α-MSH, endothelin-1, and 1,25(OH)_2_ vitamin D3 up regulate the expression of the *MC1R* gene, an effect that is expected to sustain and/or enhance the ability of melanocytes to respond to α-MSH, and thereby maintain its genomic stability and ability to synthesize the photoprotective eumelanin [63,97] (V. Swope, R. Kavanagh, and A. Abdel-Malek, unpublished work). That these paracrine factors increase *MC1R* gene expression solidifies the central role of the MC1R/α-MSH axis in regulating melanocyte survival and overcoming the genotoxic effects of UV that lead to melanoma formation. Additionally, these findings suggest that paracrine factors, via activating different receptors and signaling pathways, provide multiple means to activate DNA repair mechanisms in order to maintain the homeostasis of melanocytes and prevent the mutagenic effect of UV.

In addition to epidermal keratinocytes, dermal fibroblasts synthesize and secrete factors that regulate the activity of melanocytes. Fibroblasts in the palms and soles secrete Dikkopf1, which limits the number of melanocytes in these anatomic sites by inhibiting their proliferation and melanogenesis via suppressing β-catenin and Mitf [103]. Recently, clusterin (apoliprotien J) was reported to be secreted mainly by fibroblasts, and to inhibit melanogenesis by binding to TGFβ-1 and -2 receptors on melanocytes [104]. Neuregulin-1, a fibroblast-derived factor, increases melanogenesis by binding ErbB receptors expressed on melanocytes. Interestingly, the synthesis and secretion of neuregulin-1 by fibroblasts varied according to pigmentary phenotype, being higher in fibroblasts derived from skin phototype IV than their counterparts derived from skin phototype II [105]. These results implicate neuregulin-1 in determining constitutive pigmentation, and suggest that it contributes to eumelanin synthesis, which is abundant in dark skin. Another melanogenic factor derived from fibroblasts is CCN1/Cyr61, an extracellular matrix (ECM) protein that was recently reported to increase melanogenesis via binding to integrin α6β1 and activating the MAP kinases p38 and ERK1/2 [106]. Secretion of CCN1/Cyr61 is enhanced following UV exposure, suggesting that this factor participates in the tanning response of melanocytes to UV. Fibroblasts in young skin also secrete insulin-like growth factor (IGF)-1, which activates nucleotide excision repair in keratinocytes, thereby inhibiting UV-induced mutagenesis [107]. Melanocytes express functional IGF-1 receptors (V. Swope, R. Kavanagh, and A. Abdel-Malek, unpublished work), suggesting that IGF-1 can enhance their DNA repair capacity. Collectively, these results provide compelling evidence for the role of dermal fibroblasts and their secretome in regulating the melanocytes that reside on the basement membrane. Further studies are needed to further elucidate how fibroblast-derived paracrine factors regulate melanocytes, particularly eumelanin vs. pheomelanin synthesis and the response to solar UV.

## 7. Effect of Skin Pigmentation on Photoaging

A dramatic effect of UV exposure is photoaging, caused by long term solar UV exposure, mainly to long wavelength UVA that penetrates deep into the dermal layers, combined with intrinsic aging of skin [108]. There is clinical evidence that photoaging is more prominent and severe in light than in dark skin, and correlates directly with skin carcinogenesis [12]. This implicates low melanin (thereby eumelanin) content in sensitizing the skin to this effect of sun exposure. Photoaging results mainly from reactive oxygen species generated upon exposure to UV [109]. Exposure of fibroblasts to UVA induces the common deletion in mitochondrial DNA via ROS generation [109,110]. Mutations in the mitochondrial genome might underlie aging-associated functional changes, which include disorganization of collagen fibrils due to reduced collagen synthesis, and solar elastosis resulting from accumulation of abnormal elastin-containing material [111]. Additionally, UVA up regulates the synthesis of matrix metalloproteinases (MMPs), which degrade the extracellular matrix, including collagen. Given the role of eumelanin in reducing the sensitivity of the skin to UV by shielding it from UV rays and quenching ROS, these findings explain why lightly pigmented skin is more prone to photoaging than dark skin [9,22]. Recently, meta-analysis of genome-wide association studies in a large cohort, including 1671 twin pairs, revealed the association of SNPs at or near the pigmentary genes *SLC45A2*, *IRF4,* and *MC1R* with increased wrinkling and photoaging [12]. These results underscore the role of pigmentation in determining the extent of skin photoaging.

## 8. From the Bench to the Bedside: Selective Targeting of MC1R by Small α-MSH Analogs to Enhance Photoprotection

The well-known effect of α-MSH on stimulating eumelanin synthesis and the photoprotective effect of eumelanin have sparked interest in developing melanocortin analogs as safe sunless tanning agents. The physiological α-MSH is composed of 13 amino acids. Its small size and linear structure allowed for extensive structure-function studies, which revealed that most of the melanotropic activity of the hormone resides in the His^6^-Phe^7^-Arg^8^-Trp^9^ core sequence [112,113,114]. The best known α-MSH analog is the tridecapeptide NDP-MSH or afamelanotide. [115]. The modifications of the physiological α-MSH consisting of substitution of the fourth amino acid Methionine by Norleucine, and the seventh amino acid l-Phenylalanine by its D-enantiomer, increased the potency and stability of the resulting peptide [115,116,117]. The first clinical trial with NDP-MSH in 1991 demonstrated for the first time that injection of human volunteers with the peptide resulted in increased skin pigmentation without any sun exposure [118]. However, unexpected side effects were noted, which included loss of appetite, nausea, and flushing. These side effects are due to non-selective binding of NDP-MSH to other melanocortin receptors that were not identified yet at that time. Subsequently, systemic administration of NDP-MSH, which led to increased pigmentation, was reported to prevent sunburn and reduce UV-induced DNA damage, particularly in individuals with light skin color who burn readily upon sun exposure [119]. These reports provided compelling evidence for the efficacy of α-MSH analogs in photoprotection, and ability of melanocytes to respond to exogenous treatment with NDP-MSH, despite the synthesis of endogenous melanocortins by keratinocytes and melanocytes.

Although NDP-MSH is markedly more potent and has more prolonged effects than α-MSH, it is not selective for MC1R. Of all five melanocortin receptors (MC1-MC5R), melanocytes express only MC1R [52]. To target specifically the melanocytes with α-MSH peptide analogs, it is ideal to develop highly MC1R selective peptides to reduce off-target effects. With this in mind, we are developing small peptide α-MSH analogs that are MC1R-selective, and mimic α-MSH in enhancing DNA repair, and stimulating pigmentation. Our goal is to develop these peptides for topical application, which is more practical and provides greater target specificity than systemic administration. For topical application, peptides need to be lipophilic, in order to enhance their permeation through the stratum corneum and the epidermal layers of human skin. We have designed n-capped tetrapeptide α-MSH analogs, with 4-phenylbutyryl-His-D-Phe-Arg-Trp-NH_2_ as the lead peptide, and tested them on primary human melanocyte cultures [120]. This lead peptide proved to be considerably more potent than α-MSH in stimulating the activity of tyrosinase, hence melanogenesis, and in enhancing repair of UV-induced photoproducts, and reducing UV-induced apoptosis of human melanocytes [120]. Moreover, this peptide had a more prolonged residual effect than α-MSH on stimulation of tyrosinase activity [120]. The effects of this peptide are mediated by binding to the MC1R, as they were not evident in melanocytes expressing non-functional MC1R. Furthermore, the effects of this peptide were abolished in the concomitant presence of ASIP, the physiological MC1R antagonist. Importantly, 4-phenylbutyryl-His-D-Phe-Arg-Trp-NH_2_ is superior to NDP-MSH due to its unique selectivity for the MC1R, based on our preliminary data. Recently, Zhou et al. reported on the γ-MSH analog, [Leu^3^, Leu^7^, Phe^8^]-γ-MSH-NH_2_, as being selective for MC1R [121]. Subsequently, the same group published that replacement of Arg^8^ with Nle, and L-Phe^7^ by D-Phe in the core sequence Ac-His-Phe-Arg-Trp-NH_2_ of α-MSH conferred MC1R selectivity to the tetrapeptide [122].

We have succeeded in designing n-capped tripeptide melanocortin analogs that retain considerable melanogenic activity, despite their very small size. The lead peptide 4-phenylbutyryl-His-D-Phe-Arg-NH_2_ and other peptides with specific C-terminus modifications, were only 10 fold less potent than α-MSH in stimulating cAMP formation and tyrosinase activity of cultured human melanocytes [123]. Similar to α-MSH, these peptides were effective in inhibiting the generation of hydrogen peroxide and enhancing repair of CPD in UV-irradiated human melanocytes. As is the case of the aforementioned n-capped tetrapeptides, these tripeptides elicited their effects by binding to the MC1R, and their effects were absent in melanocytes expressing loss of function MC1R. The efficacy of these tripeptides clearly indicates that deletion of Trp^9^ does not eliminate the melanotropic activity of the tripeptides or their ability to activate the MC1R.

Our data showed that our tetra- and tripeptide analogs require expression of functional MC1R, and have no effects on cultured human melanocytes that express two loss-of-function *MC1R* variants, a genotype strongly associated with red hair, fair skin and poor tanning ability [49,120,123]. This suggests that our peptides will not benefit individuals expressing loss-of-function MC1R, who have increased UV sensitivity and high risk for skin cancer and melanoma. Others have targeted the cAMP pathway, downstream of MC1R, to circumvent the issue of loss of function of MC1R. D’Orazio et al. reported on the melanogenic effect of Forskolin on *recessive yellow* mice that express loss-of-function mutation in *mc1r* [48]. More recently, they reported on the ability of Forskolin to activate nucleotide excision repair in UV-irradiated melanocytes and melanoma cells [81]. However, Forskolin cannot be used as a tanning agent, since the cAMP pathway is promiscuous in all cell types. The limitations of our peptides do not negate the significance of utilizing selective MC1R analogs for skin cancer, including melanoma, prevention. Millions of individuals stand to benefit from this strategy, particularly those expressing mutations in other skin cancer or melanoma susceptibility genes (e.g., *CDKN2A*), and those heterozygous for *MC1R* RHC variants, who represent 50% of the entire white population in the U.S.A. [124].
